# Factors associated with the uptake of intermittent preventive treatment of malaria in pregnancy in the Bamenda health districts, Cameroon

**DOI:** 10.11604/pamj.2020.35.42.17600

**Published:** 2020-02-12

**Authors:** Ngwene Hycentha Diengou, Samuel Nambile Cumber, Claude Ngwayu Nkfusai, Mbuh Salioh Mbinyui, Vecheusi Zennobia Viyoff, Fala Bede, Lilian Akwah, Joyce Mahlako Tsoka-Gwegweni, Anchang-Kimbi Judith

**Affiliations:** 1 Department of Microbiology and Parasitology, Faculty of Science, University of Buea, Buea, Cameroon; 2 Center for Medical Research, Institute of Medical Research and Medicinal Plant studies, Ministry of Scientific Research and Innovation, Yaounde, Cameroon; 3 Faculty of Health Sciences, University of the Free State, Bloemfontein, South Africa; 4 Institute of Medicine, Department of Public Health and Community Medicine, University of Gothenburg, Gothenburg, Sweden; 5 School of Health Systems and Public Health, Faculty of Health Sciences, University of Pretoria Private Bag X323, Gezina, Pretoria, South Africa; 6 HIV Free Project, Cameroon Baptist Convention Health Services, Yaounde, Cameroon; 7 Department of Zoology and Animal Physiology, Faculty of Science, University of Buea, Buea, Cameroon

**Keywords:** Malaria, pregnancy, uptake rates, factors, Bamenda, intermittent preventive treatment

## Abstract

**Introduction:**

The World Health Organization (WHO) recommends that in malaria endemic areas with moderate to high transmission rates, pregnant women presenting for antenatal clinic (ANC) should receive at least three doses of intermittent preventive treatment in pregnancy (IPTp) for malaria between the 16^th^ and 36^th^ weeks of pregnancy at intervals of 4 weeks between doses. Several challenges remain in effective implementation of IPTp policy making the targeted coverage (80%) of the third doses of IPTp far from being achieved. The main objective of this study was to assess factors associated with the uptake of IPTp among pregnant women attending ANCs in the Bamenda Health District.

**Methods:**

To reach our objectives, we carried out a cross-sectional study following informed consent with thirty-nine (39) healthcare workers (HCW) and four hundred (400) pregnant women who were either in the third trimester of pregnancy or had recently given birth in any of thirty-six (36) health facilities (HF) within the Bamenda Health District (BHD) from May to August 2014. All sites within the BHD were included. The participants were selected by simple random sampling. The principal research instrument was a structured and pre-tested questionnaire that was designed to capture socio-demographic data and data related to stage of pregnancy and knowledge about IPTp. Data was entered using Ms Excel and analysed using SPSS v20.0. Descriptive statistics (frequencies and percentages) was used to report findings. We used Chi-Square test to compare the categorical variables (Fischer’s exact test was used in cases were conditions for Chi-Square test were not met).

**Results:**

Uptake for at least one dose of IPTp was 95.3% (381/400) and 54.9% (209/400) had received all three doses, 15.5% (59/400) received only one dose and 4.8% (19/400) did not receive any of the doses of IPTp. Knowledge about IPTp was associated with an increase uptake of IPTp (P<0.001). All health care providers were knowledgeable about the importance and use of IPTp. However, 35.9% reported not receiving any training on IPTp. Among the health providers, 28.2% did not know when to start IPTp and 43.59% did not know when to stop IPTp. Out of all the health care providers, 30.77% complained of medication (sulfadoxine-Pyrimethamine) stock out and 84.62% practiced the policy of direct observed therapy.

**Conclusion:**

The uptake of the third dose of IPTp is poor in the Bamenda Health District and this may be attributed to medication stock out and inadequacy of routine trainings for the health providers. The good practice observed was that of direct observed therapy by HCWs. Patient knowledge about IPTp in our study was associated with better uptake of IPTp. Encouraging education of pregnant women on the importance of IPTp, providing routine training to HCWs and promoting direct observation of therapy may improve on IPTp uptake during pregnancy.

## Introduction

Malaria remains a major cause of morbidity and mortality globally with significant socio-economic effects presenting a major public health challenge [1]. The World Health Organisation (WHO) reported [1] more than 207 million people developed symptomatic malaria in 2012. Each year approximately fifty million women living in malaria endemic countries throughout the world become pregnant, of whom over half live in tropical areas of Africa with moderate to high transmission rates of *P. falciparum*. An estimated ten thousand (10,000) of these women and two hundred thousand (200,000) of their infants die as a result of malaria infection during pregnancy with severe anemia from malaria contributing to more than half of these deaths [2]. Intermittent preventive treatment in pregnancy with Sulfadoxine-pyrimethamine (IPTp-SP) remains effective in preventing the adverse consequences of malaria on maternal and fetal outcome in endemic zones [3]. IPTp-SP consist of two or three doses of sulfadoxine-pyrimethamine (SP) taken at one-month interval and started in the second trimester of gestation [4] and continued until delivery [3]. Reports on IPTp uptake from studies in sub Saharan Africa show that the uptake is still below the recommended coverage set by WHO i.e. 100% by 2015 [5,6]. As shown in the Cameroon report for 2008 to 2011, 42% of women in 2008 and 32.62% of women in 2009 collected the first dose of IPT, while 28.68% and 22.68% of women collected the second dose [7]. Factors such as woman´s late booking, parity and gravidity have been shown to affect the rate of IPTp uptake [8,9]. Whereas parity, age and early booking were not associated with IPTp uptake in western rural Nigeria [10]. Attendance at ANC, financial backing, a woman´s level of education all significantly influence utilization of IPTp-SP [11,12] coupled to the opinion that knowledge about the importance of IPTp remains poor among women in developing countries. A study by Mubyazi *et al.* [13] showed understaffing, inadequate skills and poor motivation of health personnel in addition to unreliable supply of free SP at private clinics and public health facilities (HF) influence the uptake of IPTp-SP by pregnant women [13]. Prevention of malaria during pregnancy is one of the major interventions in helping to reduce maternal and infant morbidity and mortality. IPTp has proven to be efficacious in doing so, but its uptake is low [14]. The Bamenda health district report shows coverage of 84.6% for at least one dose and 54.3% for all three doses in 2013. These uptake rates are below the WHO standard of 100%. Different factors may account for this poor uptake rate of IPTp-SP in Bamenda health district (BHD). This study aimed to determine assessing of individual (pregnant woman) and service provider factors that could IPTp-SP uptake rates in BHD.

## Methods

### Study area and population

The BHD is an urban and semi-urban area with one main hospital, Bamenda Regional Hospital (BRH) that functions as the referral hospital, and many public, lay private and mission health facilities. With its roughly 337,036 inhabitants, it has 17 health areas, 36 health facilities and covers a total surface area of 560 square kilometers. The staffing levels are low compared to the staff ratio recommended by WHO. The Bamenda Health District (BHD) within the High Western Plateau of Cameroon. The weather is warm and wet most of the year. Bamenda doubles as the administrative headquarters of Mezam Division and for the northwest region of Cameroon with an estimated population of 800,000 inhabitants.

**Study population:** the study population included: 1) pregnant women at the third trimester of pregnancy; 2) postpartum women who have just put to birth and are still in the maternity; 3) health care providers working at the ANC units in any of the health facilities.

**Study design:** a cross sectional study design was used to sample the women and health providers between May and August 2014.

### Selection criteria

**Inclusion criteria:** 1) postpartum women still at the maternity; 2) women in their third trimester of pregnancy and above; 3) nurses at the ANC units visited.

**Exclusion criteria:** 1) women seeking antenatal care for the first time; 2) women within the first and second trimester.

All the health facilities in Bamenda health district were identified. The 17 health areas are made up of 36 health facilities which are subdivided into 18 public and 18 private health facilities. A public facility is a government owned facility, and a private facility is a facility owned by an individual or by a religious organization. All the facilities offering ANC services were visited on the day of their routine visits. Consent was sought from women who are in their third trimester or who had recently given birth (postpartum women) in the wards and health personnel in the various health facilities. Questionnaires were administered to consented study participants and approximately 15 minutes were used to fill the questionnaires. Those who needed help were assisted in completing the questionnaires.

### Study procedures

Administrative clearance was obtained from the Faculty of Science, University of Buea. Also, the study had administrative authorization from the Regional Delegation of Public Health in Bamenda. The authorizations were presented to all chief of centers to enable the researcher have access to the pregnant women and the health providers for the administration of the questionnaires. Consent was sought from all the pregnant women prior to the administration of the questionnaire. A structured questionnaire with closed and opened ended questions was self-administered to pregnant women and service providers (nurses and midwifes). Questionnaires were used to document socio-demographic data, ANC attendance, IPTp uptake and knowledge about ITPp-SP. Questionnaires used to collect data from healthcare workers (HCWs) assessed their understanding of IPTp-SP, its use and its implementation strategy. Poorly completed questionnaires were excluded from analysis.

**Data collection:** this was a hospital-based survey where all participants who gave consent were interviewed using a structured questionnaire filled at the hospital. Prior to its use, a total of 15 questionnaires were pretested at the University of Buea among female students aged 18 years and above with the aim of revising poorly structured questions, estimate the average time required to fill the questionnaire and thus validate the use of the questionnaire in our context. It was estimated that, each questionnaire could be administered for 30-45 minutes after the pretest. A total of 400 questionnaires were administered to pregnant women greater than or equal to 15 years of age resident in the Bamenda Health District for a period of 2 months (July-August).

### Data analysis

The data was collected on age (categorized into three categories: =20yrs, 21-25 and >25), level of education (categorized into: less than primary, secondary and higher education), parity (categorized into nullipara, primiparae and multiparae), marital status which was categorized into married or single and profession (categorized into: trader, house wife, student, civil servant).

The duration of pregnancy was measured in term of trimester of first ANC which was categorized into first trimester (0-13weeks), second trimester (14-25 weeks) and third trimester (26-37 weeks). The pregnant woman either had knowledge about IPTp or had no knowledge about IPTp. A woman was considered to be knowledgeable on IPTp, based on whether she knew why she is given drug, the number of doses and the possible outcome of not taking the drug. Each answered correctly was given a score of one or zero if incorrectly answered. A woman who scored two was said to have knowledge on IPTp and a woman who scored one or zero was considered to have no knowledge about IPTp.

The data was analyzed using SPSS version 20. Descriptive statistics (frequencies and proportions) were used to report data for categorical variable in tables and charts. Chi-Square test to compare the categorical variables (Fischer´s exact test was used in cases were conditions for Chi-Square test were not met). Means and standard deviation was used for continuous variables with normal distribution. A p-value < 0.05 was considered as statistically significant with a 95% confidence interval.

### Ethical and administrative procedures

Ethical approval for the study was sought from the Ethics Board of the Faculty of Health Sciences of the University of Buea. Informed consent was sought from each participant. Detailed explanation of the study with an opportunity given to have any doubts clarified. This was a voluntary process without any form of coercion. Participants were free to withdraw from the study at any point in time and have their information collected not used. Administrative authorization was obtained from the regional delegate of public health for the northwest region, with each hospital administrator explained the study and shown both the ethical clearance and administrative authorization prior to start of data collection.

## Results

A total of 400 women enrolled for the study participated by filling the questionnaires. The age range of the respondents was between 15-43 years; parity was para 0-6 with. Their gestational age at booking ranged from 2-38 weeks. The gestational age of the women when data was collected was between 28 and 43 weeks with a mean of 36.19 ± 4.073 (Table 1).

**Table 1:** Socio demographic characteristic of the women

Variable N=400		Urban (65.5%) n=262	Peri-Urban (34.5%) n=138	Chi-square (P-value)
Age group	≥25	52.3%	46.4%	4.210(0.1222)
	21-25	11.5%	18.8%	
	≥25	36.3%	34.8%	
Marital status	Single/widowed/divorced	21.4%	18.8%	0.356
	Married/ cohabiting	78.6%	81.2%	
Occupation	Student	21.4%	25.4%	*25.767(0.001)
	House wife	24.4%	23.2%	
	Farmer	1.9%	13.0%	
	Trader	34.7%	29.7%	
	Civil servant	17.6%	8.7%	
Educational level	≤ Primary education	23.3%	14.5%	* 9.871(0.007)
	Secondary education	52.3%	49.3%	
	Higher education	24.4%	36.2%	
Trimesters	First trimester (0-13)	4.6%	2.2%	5.490(0.064)
	Second trimester (14-25)	54.5%	71%	
	Third trimester (26-37)	40.8%	26.8%	
Parity	Nulliparous	37.4%	41.3%	0.579(0.749)
	Primiparous	28.6%	26.8%	
	Multiparous	34.0%	31.9%	
ITN use	Yes	85.5%	86.2%	3.734(0.443)
	No	14.5%	13.8%	
Religion	Christian	96.6%	92.8%	2.902(0.075)
	Non-Christian	3.4%	7.2%	

### IPTp uptake

Out of the 400 women, 381 (95.3%) reported to have taken at least one dose of IPTp-SP, 209 (54.9%) all three doses and 59 (15.5%) only one dose while 19 (4.8%) women did not receive IPTp-SP. Of the 19 who did not receive IPTp, 3 women were allergic to sulphonamides while 2 were on cotrimoxazole which are both contraindicated.

**Relationship between IPTp uptake and hospital type:** there was no significant association (p-value 0.783) between IPTp uptake and the different types of hospital as shown on.

**Relationship between age group and education with IPTp uptake:** there was no significant association between the different age groups and IPTp uptake as well as the different level of education as shown on (Table 2).

**Table 2:** General characteristics and knowledge of IPTp uptake

Variable (N=400)	IPTp uptake		p-value
Age group	Yes (%)	No (%)
≤ 20	13.2	0.8	0.365
21-25	34.2	1.5	
≥ 25	47.8	2.5	
Education			0.356
≤ Primary	25.8	2.0	
Secondary	49.2	2.0	
Higher	20.2	0.8	
Marital status			0.346
Single	19.25	1.25	
Married	76	3.5	
Parity			0.690
Nulliparous	31.75	1.5	
Primiparous	27	1.0	
Multiparous	36.5	2.25	
Trimester of first ANC(weeks)			
1^st^ trimester (0-13)	8.2	0.0	0.226
2^nd^ trimester (14-25)	76.0	3.8	
3^rd^ trimester (26-37)	11.0	1.0	
Religion			0.001
Christians	91.0	4.2	
Non-Christians	4.2	0.5	
Knowledge on IPTp			< 0.001
Knowledgeable	56.0	0.0	
No knowledge	39.2	4.8	

**Marital status and parity versus IPT uptake:** there was no significant difference in the uptake rates between the married and single women as well as no difference in uptakes between the different parous groups as shown on (Table 2).

**Relationship between trimester of first ANC and occupation with IPTp uptake:** there was no significant association between trimester of first ANC and occupation with IPTp uptake (Table 2).

### Pregnant women´s knowledge and religion versus IPT uptake

The religion of the pregnant woman was not associated with the uptake of IPT as shown on the table below. The pregnant women knowledge is significantly associated with the uptake of IPT. Out of all the women who took IPT, only 166 knew why they were given the drug and 113 had no idea why they were being giving the drug and the others had different reasons why they taught they were being given the drug. Of all the women, 191 knew the recommended number of doses that is three doses while 145 had no idea about the number of doses to be given while the rest had different number of doses ranging between one and four and every clinic visit. Also, 235 women knew of different consequences of not taking IPT and 146 had no idea about the consequences of not taking the drug. The summary of the knowledge is presented on (Table 2).

**Practice of direct observed therapy (DOT):** of all the women, 254 (66.7%) took the drug in front of the health provider while 127 (33.3%) did not take the drug in front of the provider. There was no significant difference between those who practice DOT and those who did not (P=0.472).

**Health provider factors associated with IPT uptake:** a total of 39 health providers participated in the study which was made up of 34 females and 5 males. Their ages ranged between 22 and 50 with a mean and standard deviation of 34.51 ± 8.236. All the nurses knew the number of doses of IPTp which are supposed to be given to pregnant women.

### Frequency of possible health provider factors associated with IPT

**Qualification of health providers:** the nurses had qualifications which ranged from first school leaving certificates to degrees. Most of the nurses were state registered nurses. About 10% of the "nurses" had no formal training in nursing practice.

**Training on IPT:** out of the 39 health providers, 64.1% (25) have received training on IPTp while 35.9% (14) health providers reported no training on IPTp.

**Figure 1:**
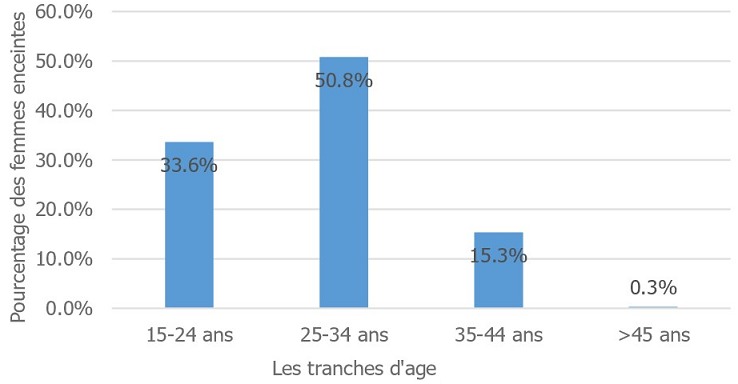
Knowledge on timing of first ITPp

**Figure 2:**
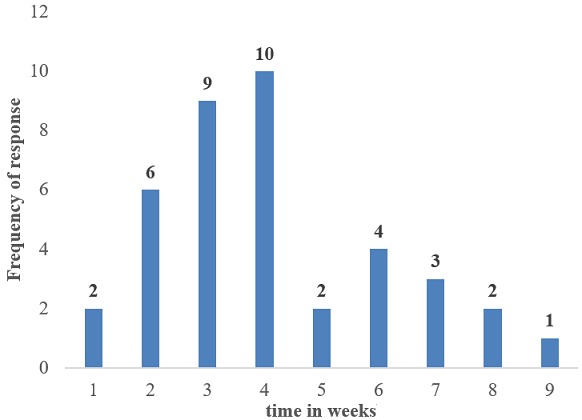
Knowledge on when to stop IPTp

**Knowledge on timing of IPTp:** out of the 39 health providers, 71.8% (28) health providers knew when to start giving IPTp i.e. between 16 and 18 weeks while 28.2% (11) did not know when to start giving IPTp as shown on the bar chart below. Also, 41.0% (16) health providers did not know exactly when to stop IPTp and 59% (23) knew when to stop IPTp as shown on (Figure 1, Figure 2).

**Drug supply:** out of all the health providers, 30.77% (12) reported on irregular supply of drugs while 69.23% (27) have regular supply of drugs and the different approaches used when there is shortage of drugs.

**Figure 3:**
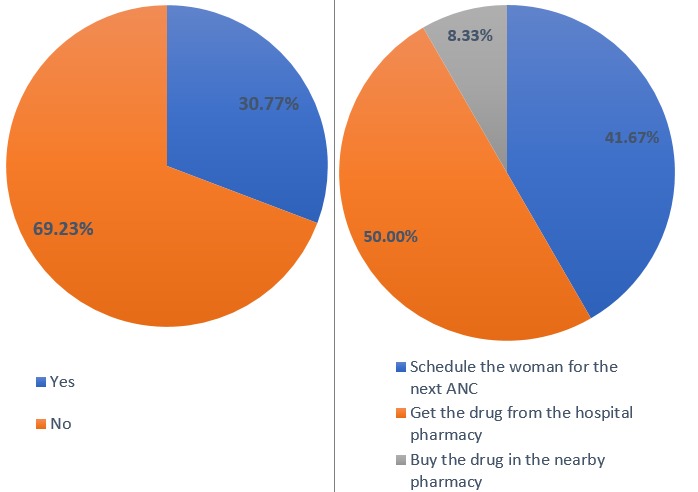
Supply of IPTp: A) regular supply of drug; B) things done when stock is finish

**Figure 4:**
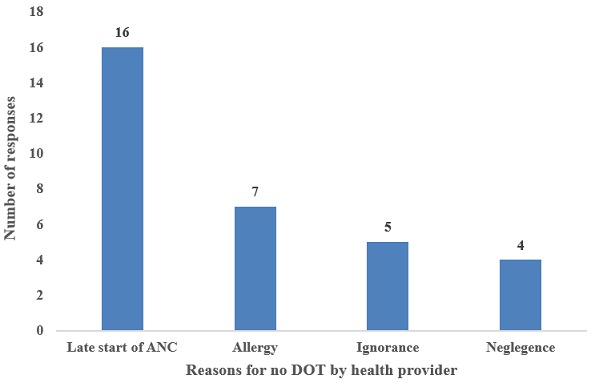
Reasons why DOT is not practice by some health providers

**Practice of DOT:** DOT is a common practice among health providers in the Bamenda health district. Out of the 39 health providers, 15.38% (6) health providers did not practice the DOT approach and the reasons they presented are shown in the Figure 3. Some of the reasons given by the nurses why they don´t practice the policy of DOT are shown in (Figure 4).

**Figure 5:**
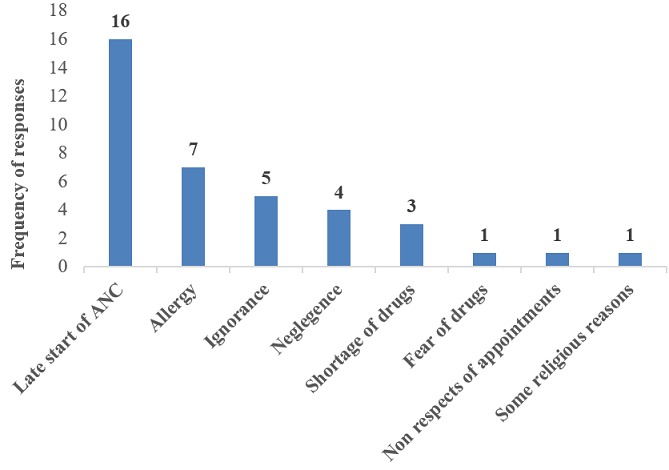
Health provider’s reasons on why some women refuse to take IPTp-SP

**Health provider´s reasons on why some women refuse to take IPTp-SP:** most of the health providers attributed late ANC attendance to why women don´t take the complete dose of IPTp followed by the allergic reactions of the drugs and others as shown on (Figure 5).

## Discussion

The study showed that IPTp uptake for at least one dose was 95.3% which was similar to studies by Anchang-Kimbi *et al.* [15] that showed uptake of 90% in Mutengene. Among those who took IPTp, 54.9% received all three doses. This uptake rate of all three doses is similar to what was observed in the Bamenda Health District report for 2013 (54.33%). The uptake of 95.3% for at least one dose seen in this study is slightly above 84.60% that was reported in the Bamenda Health District (Bamenda Health District Report for Malaria, 2013). These rates were similar to what was observed in Malawi [16]. These uptake rates in Bamenda can be attributed to the fact that most of the health facilities practice the policy of direct observed therapy. This is corroborated by our findings, 254/400 (66.7%) reported to have taken the drug under direct observation by a health provider. The policy of DOT was affected negatively by lack of water and cups in some health facilities. Some women also said they will prefer to take the drugs at home after meals. Another factor which enhanced the uptake rates of IPTp in Bamenda health district was the health education given to the women at each ANC visit. These health talks created awareness for the women who will be more likely to request for IPTp if HCW forgot.

Knowledge on IPTp was statistically significantly associated with increase uptake of IPTp among pregnant women in our study. Whereas factors such as parity, marital status, religion, educational level, trimester of first ANC and occupation were not statistically significantly associated with the uptake of IPTp in the BHD. Studies carried out by Takem *et al.* [8], and Amon *et al.* [10] and a systematic review by Holtz *et al.* [17] demonstrated knowledge about IPTp was a predictor of the uptake of IPTp which is in line with the findings of our study. This high uptake rates in women who are knowledgeable about IPTp, can be attributed to the fact that awareness about the drug and the consequences of not taking the drug will make the woman request for the drug in case the health care provider forgets.

WHO recommends that all women should receive the first dose of IPTp as early as possible after quickening (first movements of the fetus) at about 16 weeks; health providers should give three doses of SP under direct observation to women reporting for ANC. Health provider should give each dose at least one month apart with every schedule visit to the clinic. WHO recommends at least four ANC visits for every uncomplicated pregnancy? Providers can administer the drug to the women either in an empty stomach or with food [3]. The national policy in Cameroon states that women should be given three doses of SP within the 16^th^ and the 36^th^ weeks of pregnancy at intervals of 4 weeks between doses [7].

Studies carried out by Olliaro *et al.* [18] shows that the quality of health services provided influences the rate of IPTp uptake. Of the 39 health providers interviewed, all could correctly tell the medication used for IPTp, they understood the reasons for the administration of the SP during pregnancy and the number of doses to be given. Of all the health providers, 28.2% did not know when to start IPTp, 41.0% did not know exactly when to stop IPTp and 35.9% reported not receiving training on IPTp. Ten percent (10%) of the health providers were not qualified since they have not had any formal training in nursing. Studies carried out by Ouma *et al.* [19] showed an increase in uptake after nurses were trained. There have been reports of pregnant women not taking IPTp because the HCWs were not aware of the IPTp guidelines [20]. In addition, limited opportunities for training and career development among staffs negatively influences the uptake of IPTp [13]. If all the health providers in BHD were trained, there is likely to be an increase in uptake rates of IPTp-SP. This is because the health providers will be familiar with the IPTp guidelines, thereby increasing their knowledge, that of the pregnant woman.

Healthcare providers, 30.77%, reported stock-outs which directly influence the uptake of IPTp. As women may have reported for ANC and there was no medicine (SP). In such cases HCWs were usually intuitive to give an appointment to such pregnant women. However, the women were more likely not to respect such appointments. Some other reasons advanced by HCWs why women presenting for ANC did not get IPTp-SP were: lack of water or cups, and some women preferring to not take medicine without meals (despite the fact that it was not an absolute contra-indication). Some HCWs admitted preferring to advise pregnant women to take IPTp at bed time so as to “minimize the side effects of the drugs”. HCWs reported late start of ANC as one of the reasons why it is difficult for some women to receive the number of recommended three doses of IPTp-SP.

## Conclusion

Uptake of IPTp for at least one dose is high (95.9%) whereas uptake of the recommended three doses for IPTp is still low. Pregnant woman´s knowledge on IPTp was the only factor significantly associated with IPTp uptake in the study. DOT is effective in Bamenda health district as most health providers and women practice direct observe therapy.

### What is known about this topic

- Factors influencing the uptake of intermittent preventive treatment of malaria in pregnancy;
- A survey of knowledge, attitude and practice of malaria management among pregnant women;
- Safety, efficacy and determinants of effectiveness of anti-malarial drugs during pregnancy: implications for prevention programs in *Plasmodium falciparum* endemic.

### What this study adds

- The uptake of IPTp for at least one dose is high (95.9 %) whereas uptake of the recommended three doses for IPTp is still low in the Bamenda Health District, Cameroon;
- Pregnant woman’s knowledge on IPTp was the only factor significantly associated with IPTp uptake in the study. DOT is effective in Bamenda health district as most health providers and women practice direct observe therapy;
- There is a high IPTp-SP uptake rate in Bamenda Health District, Cameroon.
